# Disclosure of diagnosis – Adherence – Couple or future of the past

**DOI:** 10.1186/1471-2334-14-S7-O6

**Published:** 2014-10-15

**Authors:** Odette Chirilă, Sorin Petrea

**Affiliations:** 1 National Institute for Infectious Diseases "Prof. Dr. Matei Balş", Bucharest, Romania

## Background

HIV infection causes in the patient the development of specific attitudes toward diagnosis, leading even to a psychological change in the patient's personality expressed by a certain type of mental, emotional and behavioral participation, variable time depending on the stage of infection. The children of the Romanian cohort have an emotional and behavioral baggage marked by depression, anxiety, adjustment disorder, acquired a pronounced psychological fragility, with major impact in medical condition, which means non-adherence, suicide attempts etc. The disclosure of diagnosis (DD), adherence and desire of a family are defining notes describing these patients.

## Methods

Psychological interventions allow a change in patterns of thinking and behavior of patients, by unlearning negative experiences and relearning of the experiences with positive value. Therefore, counseling and psychotherapy are methods whereby the HIV patient identifies alternative behaviors that learn to operate adapted biological and psychological age. DD was a first stage in caring of HIV children, aimed at the understanding and acceptance of the experiences related to HIV diagnosis. Regarding children, adherence depends significantly on the relationship with the adult, parents, on the one hand and the care team on the other hand. If the HIV infected child had no adherence issues, the adolescent with HIV had serious adherence problems, both because of the fragility of age, but especially because of therapeutic fatigue. The age of young HIV adult brings forward the couple relationship, when all behaviors come early: love, desire for starting a family, the child.

## Results

DD determined changing of the personal beliefs associated with a diagnosis perceived as serious, leading to living with HIV diagnosis, through a permanent redefinition of their limits and psychological resources. DD removed the distorted perception of this diagnosis, which would have led to a defensive attitude to disease and thus would have compromises adherence. The HIV patients who accept the diagnosis have a good adherence, those who deny the diagnosis have a compromised adherence and those who resign, are not interested about their own life, have a very poor adherence. The couple patterns which are adjusted, functional and congruent have significantly influence on the adherence of each partner.

## Conclusion

An arch over time proves that the defining landmarks in the 20 year-old patient are found in the course of their childhood: who and how will disclose the HIV diagnosis, theirs or their parents’, repeated experiences of their parents? History will become the future?

